# A Neovascularized Left Atrial Mass

**DOI:** 10.1155/2012/518539

**Published:** 2012-04-18

**Authors:** Rania Hammami, Leila Abid, Souad Mallek, Ilyes Kharrat, Mariem Ellouz, Mourad Hentati, Imed Frikha, Samir Kammoun

**Affiliations:** ^1^Cardiology Department, Hédi Chaker University-Hospital, 3029 Sfax, Tunisia; ^2^Cardiovascular Surgery Department, Habib Bourguiba Hospital, 3029 Sfax, Tunisia

## Abstract

*Background*. The discovery of a large left atrial mass through echocardiography obliges the clinician to perform a differential diagnosis to distinguish tumor from thrombus. The neovascularization of the mass could be helpful to predict the type of the malformation and whether it is in favour of a vacular tumour rather than a thrombus . *Observation*. A 43-years-old man who had no cardiac antecedent reported that he have had dyspnea and palpitation since 10 months. The cardiac auscultation, revealed an irregular rhythm with diastolic murmur at the apex. The electrocardiogram showed an atrial fibrillation. The transthoracic echocardiography revealed a severe mitral stenosis with a huge left atrial mass, confirmed through transesophageal echocardiography. After 4 weeks of an efficient anticoagulant treatment, the mass was still persistent in the echocardiography. So we decided to resect the mass and to achieve a mitral valve replacement. The preoperative coronarography showed neovascularization among the mass and fistula from the circumflex artery. Considering the characteristic of the mass (neovascularization and resistance to anticoagulant), we strongly suspected a vascular tumor especially myxoma, but the histological exam revealed an organized thrombus. *Conclusion*. Coronary neovascularization is a specific sign for left atrial thrombus in mitral stenosis, but surgery is the best way to confirm diagnosis.

## 1. Background

The discovery of a large left atrial mass through echocardiography obliges the clinician to perform a differential diagnosis to distinguish tumor from thrombus. In fact, magnetic resonance imagery could be useful to identify the mass but it couldnot distinguish tumor from organized thrombus. Certainly, surgery is the best solution for a successful diagnosis, but what about angiographic coronary? Does it play any role in the recognition of this mass?

## 2. Observation

A 43-year-old man who had no definite history of rheumatic fever was admitted in our department in October 2008, for dyspnea and palpitation evolving since 10 months. Physical examination revealed an irregular pulse, at a rate of 100 beats/min, and polypnea at 30 cycles/min. A prominent diastolic murmur continuing to the first sound was noted at the apex. The electrocardiogram revealed an atrial fibrillation with a right bundle block. The chest X-ray film showed an enlarged left atrium and a pulmonary congestion.

**Figure 1:**
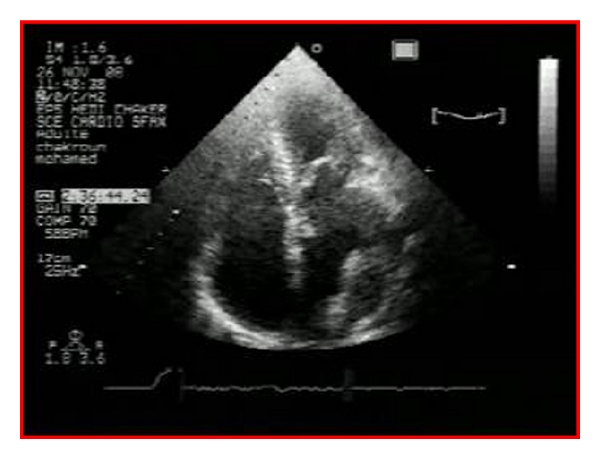
Transthoracic echocardiography: 4 chambers view showing a well-circumscribed mass in the let atrium.

**Figure 2:**
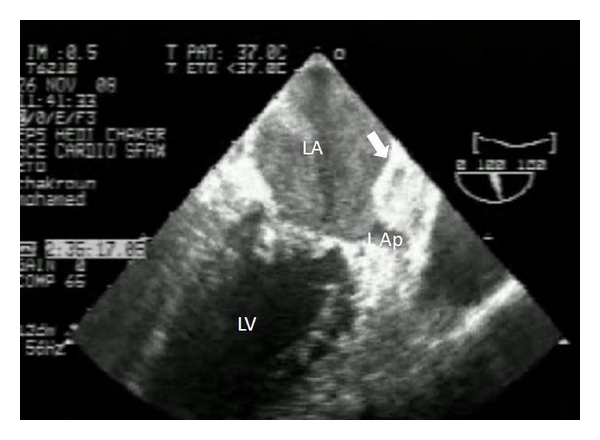
Transesophageal echocardiography showing an important spontaneous contrast and a large mass (white arrow) in both left atrium and appendage (LA: left atrium; LV: left ventricle; L Ap: Left appendage).

**Figure 3:**
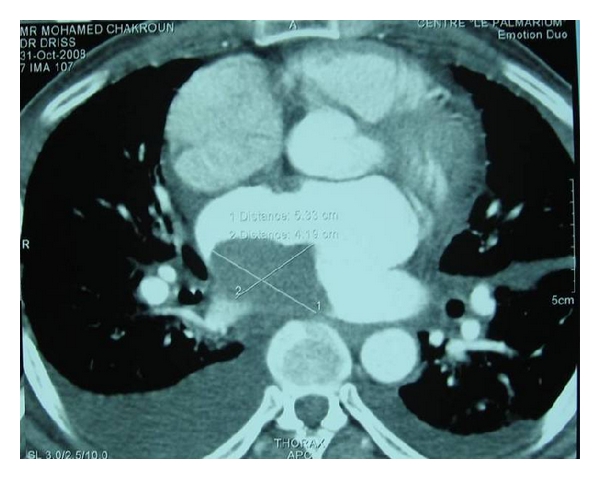
The transthoracic tomography showing a left atrium tissular mass in the left atrium.

**Figure 4:**
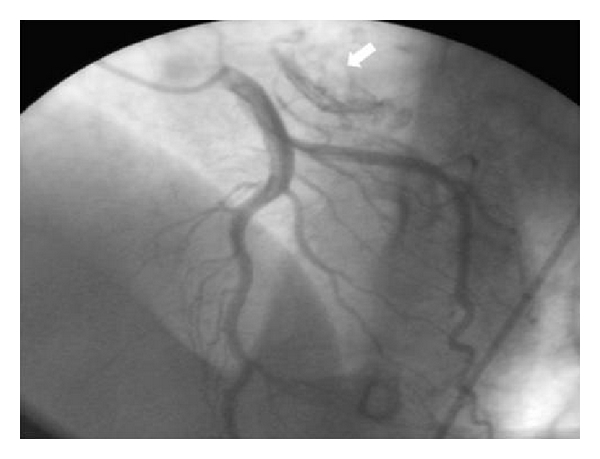
Angiographic coronary showing the neovascularization and the fistula.

**Figure 5:**
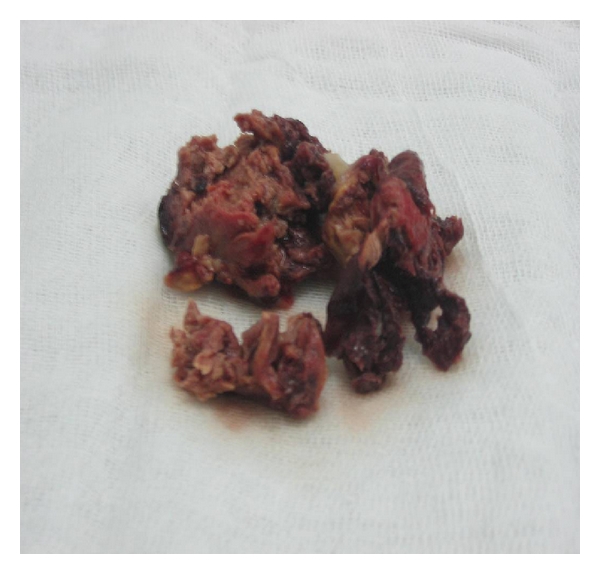
Surgery findings: left atrial mass which seems to be friable and irregular.

A transthoracic echocardiography was performed (Figure 1) and had showed a critical mitral stenosis, with a mitral valve area of 0,7 cm^2^ by planimetry and 1 cm^2^ by pressure half time. The peak/mean gradients across the mitral valve were 16/8 and there was mild regurgitation. There were severe pulmonary arterial hypertension (70 mmHg) and mild functional tricuspid regurgitation. The mitral valve appeared rheumatically deformed, with moderately thickened leaflets, and commissural fusion, but there were calcifications. Other valves were normal. The left atrium was enlarged with an area of 40 cm^2^. A huge intra-atrial mass was apparent on two-dimensional mode echocardiography. This mass was well circumscribed, surfacing 10 cm^2^, heterogeneous, and sticking to the atrial wall. On transesophageal echocardiography (Figure 2), we found that the mass was located on a huge left atrial appendage and extended to the left atrium. Moreover, this one contained an important spontaneous contrast. The subvalvular chordae were thickened and shortcut but without calcifications. With the presence of severe mitral stenosis, an enlarged atrium, and an atrial fibrillation, we thought that it was a thrombus. So, the patient had benefited from an efficient anticoagulant treatment for 4 weeks. But, on the echocardiographic control, we noted the persistence of the mass. A transthoracic tomography was performed to identify the mass limits and connections (Figure 3). It showed the presence of a tissular mass in the left atrium, measuring 6 cm of long axis, with an ovular form and regular borderlines. Therefore, a surgical resection of the mass and a mitral valve replacement were indicated. Because our patient was aged more than 40, an angiographic coronary was done before surgery. There werenot coronary lesions but we discovered neovascularizations arising from a branch of the left circumflex artery (LCX) with fistula formation manifested by a dense mass stain and squiring of contrast material into the left atrial cavity (Figure 4). Considering the clinical and paraclinical data, we suggested two diagnoses: a thrombus or a vascular tumor mainly myxoma since it was ovular, well circumscribed, and located on the left atrium. Given the systemic embolism risk, a decision to carry out an emergency operation was taken. The surgery was accomplished through a conventional median sternotomy and full cardiopulmonary bypass with ascending aortic perfusion and bicaval drainages. A standard left atrial incision through the interatrial groove was performed after aortic clamping. The mass has a dark red colour and it was enucleated and friable. It measured 65 mm *30 mm (Figure 5). The fistula opened at the atrium and raised from circumflex artery. So, it was sutured. The mitral valve was replaced with mechanic prosthesis. Mitral valve repair was not undertaken because of excessive leaflet thickening and shortness of chordae to both the anterior and posterior leaflets. Postoperatively, the patient made an uncomplicated recovery. The histological exam led to the conclusion that there was an organized thrombus associated with rheumatic mitral disease.

## 3. Discussion

Thrombosis within the left atrium is a common place phenomenon in rheumatic heart disease. Its genesis is influenced by the type of mitral valve dysfunction, dilatation of the left atrium, and the presence of atrial fibrillation.

Detection of such thrombi by transthoracic or trans-esophageal echocardiography is very specific (99%) [1] and important as they are potential sources of thromboemboli. In fact, the treatment is based on heparin treatment associated with aspirin for at least 4 weeks. Surgical resection would be indicated if an efficient anticoagulant treatment fails. But, in our case, we were not sure that it was a thrombus and not a tumor.

Myxoma is the main differential diagnosis suggested for our patient as we know that this kind of tumor is located in 90% on left atrium. It is in general well circumscribed and has an ovular form. Moreover, angiographic demonstration of abnormal vessels arising from the coronary arteries has been for long time reported as being an isolated congenital cardiac anomaly, concomitant with cardiac tumors, especially myxomas [2].

The magnetic resonance imaging (MRI) has an important contribution in the differentiation of thrombus from myxoma. After gadolinium injection, myxoma is lifting up on periphery whereas thrombus remains unchanged. However, thrombus could also lift up on periphery when it is organized [3, 4].

 Angiographic coronary could be helpful to make diagnosis. On literature, many studies suggested that neovascularity and fistula formation from coronary arteries to the left atrium were usually associated with organized atrial thrombosis in patients with mitral valve disease.

The first publication was reported by Marshall Jr. et al. in 1965 [5]. He described a “tumor vascularity” demonstrated during selective coronary angiography in a patient with unsuspected left atrial myxoma. Other authors observed similar findings in a patient with mitral stenosis and severe coronary atherosclerosis. Although a large left atrial thrombus was found at operation, the presence of neovascularity was at that time, related not to the thrombus but to coronary artery disease. Standen used selective coronary angiography in 1975 [6] and he described “tumor vascularity” as abnormal vessels arising from the left circumflex artery to the left atrium in a patient with severe mitral stenosis. A left atrial thrombus was found at surgery.

Colman et al. in 1981 [7] had performed a retrospective study including 507 patients with mitral valve diseases. The preoperative coronary angiograms were reviewed. Atrial thrombosis was present in 15% (76 patients). Among 30 patients having angiographic neovascularity and fistula formation, the thrombi were always arising from the circumflex coronary artery. These coronary findings had a specificity of 98,8% and a sensitivity of 33% for the diagnosis of thrombus in left atrium. No relation was found between these signs and the size and the histologic age of the thrombi. Morgan et al. [8] had retrospectively enrolled 75 patients with severe mitral stenosis between 1984 and 1986. They reviewed echocardiograms and coronarograms before mitral valve replacement. They showed that coalition of left atrial mass on echocardiography to neovascularization on coronarography is specific of thrombosis (99-100%).

The mechanism of fistula and neovascularization is not clear. Standen [6] suggested that the fistula formation in the left atrium resulted from partial necrosis of the organized thrombus with ulcerated surface, which allowed coronary blood to escape into the left atrial cavity. However, Colman et al. [7] found no correlation between neovascularization with fistula formation and histologic findings of left atrial thrombi.

## 4. Conclusion

Considering this case report, the management of atrial mass will be based first on the clinical history (mitral stenosis, permanent atrial fibrillation evaluating since many years, etc) and the echocardiography (well-limited mass, echogenicity, etc). When we suspect a thrombus, a treatment test by anticoagulation could be very helpful to confirm the diagnosis.

In atrial mass persistent despite an efficient anticoagulation, we can perform either RMN or coronarography. After gadolinium injection in RMN, myxoma is lifting up on periphery whereas thrombus remains unchanged. However, thrombus could also lift up on periphery when it is organized. But during coronarography, we can easily conclude to thrombus when we discovered a neovascularization of the mass.

Finally, surgery remains the best option to confirm diagnosis and to treat the mass.
